# Development, External Validation, and Visualization of Machine Learning Models for Predicting Occurrence of Acute Kidney Injury after Cardiac Surgery

**DOI:** 10.31083/j.rcm2408229

**Published:** 2023-08-09

**Authors:** Jiakang Shao, Feng Liu, Shuaifei Ji, Chao Song, Yan Ma, Ming Shen, Yuntian Sun, Siming Zhu, Yilong Guo, Bing Liu, Yuanbin Wu, Handai Qin, Shengwei Lai, Yunlong Fan

**Affiliations:** ^1^Medical School of Chinese PLA, 100853 Beijing, China; ^2^Department of Vascular and Endovascular Surgery, The First Medical Center of Chinese PLA General Hospital, 100853 Beijing, China; ^3^Department of Cardiovascular Surgery, the First Medical Centre of Chinese PLA General Hospital, 100853 Beijing, China; ^4^Department of Cardiovascular Medicine, The First Hospital of Hebei Medical University, 050000 Shijiazhuang, Hebei, China; ^5^Department of Cardiovascular Surgery, the Sixth Medical Centre of Chinese PLA General Hospital, 100048 Beijing, China

**Keywords:** acute kidney injury, cardiac surgery, machine learning, prediction model, precision medicine

## Abstract

**Background:**

Cardiac surgery-associated acute kidney injury (CSA-AKI) is a major complication that results in short- and long-term mortality among patients. Here, we adopted machine learning algorithms to build prediction models with the overarching goal of identifying patients who are at a high risk of such unfavorable kidney outcomes.

**Methods:**

A total of 1686 patients (development cohort) and 422 patients (validation cohort), with 126 pre- and intra-operative variables, were recruited from the First Medical Centre and the Sixth Medical Centre of Chinese PLA General Hospital in Beijing, China, respectively. Analyses were performed using six machine learning techniques, namely K-nearest neighbor, logistic regression, decision tree, random forest (RF), support vector machine, and neural network, and the APPROACH score, a previously established risk score for CSA-AKI. For model tuning, optimal hyperparameter was achieved by using GridSearch with 5-fold cross-validation from the scikit-learn library. Model performance was externally assessed via the receiver operating characteristic (ROC) and decision curve analysis (DCA). Explainable machine learning was performed using the Python SHapley Additive exPlanation (SHAP) package and Seaborn library, which allow the calculation of marginal contributory SHAP value.

**Results:**

637 patients (30.2%) developed CSA-AKI within seven days after surgery. In the external validation, the RF classifier exhibited the best performance among the six machine learning techniques, as shown by the ROC curve and DCA, while the traditional APPROACH risk score showed a relatively poor performance. Further analysis found no specific causative factor contributing to the development of CSA-AKI; rather, the development of CSA-AKI appeared to be a complex process resulting from a complex interplay of multiple risk factors. The SHAP summary plot illustrated the positive or negative contribution of RF-top 20 variables and extrapolated risk of developing CSA-AKI at individual levels. The Seaborn library showed the effect of each single feature on the model output of the RF prediction.

**Conclusions:**

Efficient machine learning approaches were successfully established to predict patients with a high probability of developing acute kidney injury after cardiac surgery. These findings are expected to help clinicians to optimize treatment strategies and minimize postoperative complications.

**Clinical Trial Registration:**

The study protocol was registered at the ClinicalTrials Registration System (https://www.clinicaltrials.gov/, #NCT04966598) on July 26, 2021.

## 1. Introduction

Cardiac surgery-associated acute kidney injury (CSA-AKI), with an incidence ranging from 8.9 to 39.0%, is a common and serious health complication [1, 2]. Any, even subtle, changes in renal functions are associated with late survival outcomes [3]. To properly manage CSA-AKI, several risk scores have been developed using multivariable logistic regression analysis [4, 5, 6, 7, 8]. However, all risk models based on logistic regression methods are limited by the statistical assumption of linear inherence [9]. In addition, due to challenges associated with overfitting and multi-collinearity of the logistic regression analysis, only a handful of input variables have been analyzed. This calls for identification of advanced algorithms to promote the development of more flexible and efficient models for predicting AKI. Machine learning, an effective computer algorithm that deals with multidimensional data analysis, has been extensively applied in medicine to solve medical problems [10, 11]. Machine learning has numerous functions, including risk stratification [12], diagnosis and classification [13], and survival predictions [14], which makes it applicable to many tasks.

In this study, various machine learning techniques were employed to develop models for predicting CSA-AKI [15, 16, 17, 18, 19]. This study differs from previous studies using machine learning to predict CSA-AKI in several key ways: (i) The predictive performance of established models was compared to that of an existing risk model derived from conventional logistic regression analysis, the APPROACH score [6]. (ii) This is a multi-centre study in which the model was externally validated, and the discriminatory power and clinical net benefit of the model were externally assessed using receiver operating characteristic (ROC) curves and decision curve analysis (DCA) curves, respectively; (iii) In-depth visualization of the model was performed, which not only revealed individual-level predictive evidence but also the impact of different variables on predicted outcomes. This partially revealed important insights into the power of models that enable machine learning.

## 2. Materials and Methods

### 2.1 Study Population

Data of patients who underwent adult cardiac surgery between January 2017 and December 2020 at two medical centers, namely the First Medical Centre and the Sixth Medical Centre of Chinese PLA General Hospital, Beijing, China, was retrieved for analysis. The Institutional Review Board of Chinese PLA General Hospital approved this study. Due to the observational nature of the study, the requirement for an informed consent was waived. This study followed the Transparent Reporting of prediction model development and validation for Individual Prognosis Or Diagnosis (TRIPOD) statement.

**Fig. 1.**
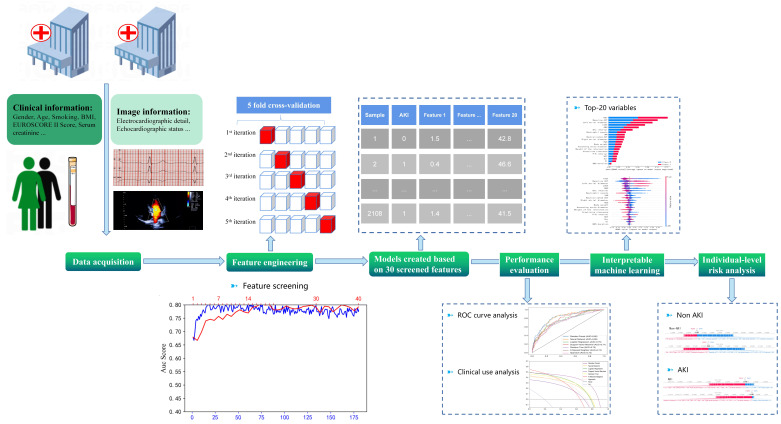
**A schematic representation of the experimental framework**. The entire dataset comprised 2108 patients from two medical centres. After feature engineering, six machine learning algorithms were employed to build the model. In the external test set, receiver operating characteristic (ROC) curve was generated and used to determine discrimination across the six models, whereas decision curve analysis (DCA) was performed to determine the models’ net benefit in clinical practice. Finally, the models were visualized to reveal the key areas driving the predictions. AKI, acute kidney injury; BMI, body mass index; EuroSCORE II, European System for Cardiac Operative Risk Evaluation II.

An overview of the experimental procedures used in this study is shown in Fig. 1. A total of 1686 patients from the First Medical Centre of Chinese PLA General Hospital were screened for model development, while 422 others from the Sixth Medical Centre of Chinese PLA General Hospital were used for external validation. For greater generalizability to real-world contexts, candidates were screened using minimal exclusion criteria, excluding only patients under 18 years of age, as these groups were exempted from the European System for Cardiac Operative Risk Evaluation (EuroSCORE) II calculator.

### 2.2 Data Collection

The electronic health records of two medical centers were retrieved and used to create datasets that included 126 preoperative and intraoperative variables. Preoperative data included patients’ demographic characteristics, medical and medication histories, as well as baseline laboratory findings. Intraoperative variables were extracted from the cardiopulmonary bypass records and the anesthesia information management system. The Euroscore II (http://www.euroscore.org/calc.html) and creatinine clearance were calculated for each patient, with the latter obtained using the following formula:

Creatinine clearance (mL/min) = (140 – age (years)) × weight (kg) × (0.85 if female) / [72 × serum creatinine (mg/dL)]

Notably, AKI was the primary endpoint and was based on the 2012 Kidney Disease: Improving Global Outcomes (KDIGO) criteria (https://kdigo.org/conferences/nomenclature/), which refers to the maximal change in serum creatinine during the first seven days after the operation. Specifically, AKI was diagnosed either when postoperative serum creatinine level was 1.5-fold greater than at baseline or when an increase in serum creatinine of 0.3 mg/dL occurred within 48 h postoperatively. Baseline serum creatinine levels were the preoperative serum creatinine values that were closest to the time of surgery.

### 2.3 Data Processing

Prior to analysis, data were preprocessed using the following approaches: (i) Data cleaning was performed to identify any missing values, outliers, and duplicates. Missing values were observed in <5% of the records. All cases with missing data were excluded during modeling, and no missing data imputation was performed. (ii) Features were extracted by generating extra variables based on existing ones, such as body mass index (kg/$m^{2}$), blood loss (mL/kg), and urine output (mL/kg). Feature selection, which is the automatic selection process of the most relevant feature subset to the outcome event (removal of irrelevant, weakly relevant or redundant features), was ill-suited during model development because all features were enrolled and further screened for analysis in the nested models (see below). Prior to modeling, both continuous variables and classified variables were subjected to standardization and One-Hot Encoding, respectively. In addition, the Synthetic Minority Over-sampling Technique (SMOTE) algorithm was applied to address potential imbalances in the training dataset.

The entire study dataset consisted of a training set comprising 1686 patients from the First Medical Centre of Chinese PLA General Hospital, and a testing set made up of 422 cases from the Sixth Medical Centre of Chinese PLA General Hospital. Model development was achieved by using different machine learning approaches within training datasets. For model tuning, hyperparameters were optimized for each classifier using the GridSearch method in a 5-fold stratified cross-validation on the training dataset. Further external validation was performed using the testing dataset to determine the model’s predictive performance and to visualize the key areas influencing predictions. The statistical significance of differences between area under the curves (AUCs) was tested using the DeLong test [20].

### 2.4 Machine Learning Techniques

The prime and most mature machine learning methods, including K-nearest neighbor, logistic regression, decision tree, random forest, support vector machine, and neural network were employed to predict CSA-AKI events. The prediction performance of these nonlinear models was compared to that of a traditional linear model: the APPROACH risk score. Briefly, the nested models were assembled by adding each of the 126 variables in order of increased ranking in variable importance during modeling. Next, predictive accuracy of the nested models was determined by generating AUC of the ROC. These were used to identify the best-performing model and the cut-off threshold values in all variables. Among the six nested models, AUC scores of the random forest (RF) classifier had peaked at about 20 variables; therefore, this was selected as the cut-off value. The six models were further re-assembled based on variables that contributed the most, that is, the top 20 variables based on the RF classifier. Next, ROC and DCA plots were used to externally validated performance of re-integrated models using the external dataset. The AUC score of ROC curve was measured to show model discrimination, while the DCA assessed the net benefit of clinical utility. Furthermore, interpretable machine learning in the best classifier, the RF model, was provided via a visual explanation AI program: the SHapley Additive exPlanation (SHAP) package. The resulting SHAP value was used to quantify each variable’s contribution to the impact on the model output and further explain the accountable predictive outcomes. In addition, the Seaborn library approach was adopted for data visualization to elucidate the interplay among different variables as well as between variables and outcomes.

### 2.5 Statistical Analysis

Data analyses were performed using packages implemented in Python Software (version 3.6, Python Software Foundation, Wilmington, DE, USA) and Scikit-learn (https://scikit-learn.org/). The resulting data were visualized using SHAP values and the Seaborn library (https://seaborn.pydata.org/index.html). Descriptive statistics were presented as medians (interquartile ranges) or numbers (percentage). The Mann–Whitney U test, Pearson chi-square test, or Fisher exact test were used for analyses, as appropriate.

## 3. Results

### 3.1 Patient Characteristics

A total of 637 (30.2%) out of the 2108 participants underwent CSA-AKI attack within seven postoperative days. Incidence of CSA-AKI stages 1, 2, and 3 were 20.2% (426/2108), 4.7% (99/2108), and 5.3% (112/2108), respectively. Comparisons of the relevant data between patients who developed AKI and those who did not is outlined in **Supplementary Table 1**. The results showed statistically significant differences in clinical variables between the groups (*p* < 0.05). Specifically, in **Supplementary Table 1**, patients who developed AKI were relatively older and had higher Euroscore II scores compared to those who did not. In addition, poor cardiac conditions (New York Heart Association (NYHA) Functional Classification, left ventricular ejection fraction (LVEF)) were associated with a substantially higher risk of CSA-AKI. Dyslipidemia, diabetes, and hypertension also increased the risk of AKI. Besides, other biochemical risk factors include anemia, hypoalbuminemia, and thrombocytopenia. However, coronary angiography failed to find a significant association with AKI.

### 3.2 Prediction Models

#### 3.2.1 Nested Models

**Fig. 2.**
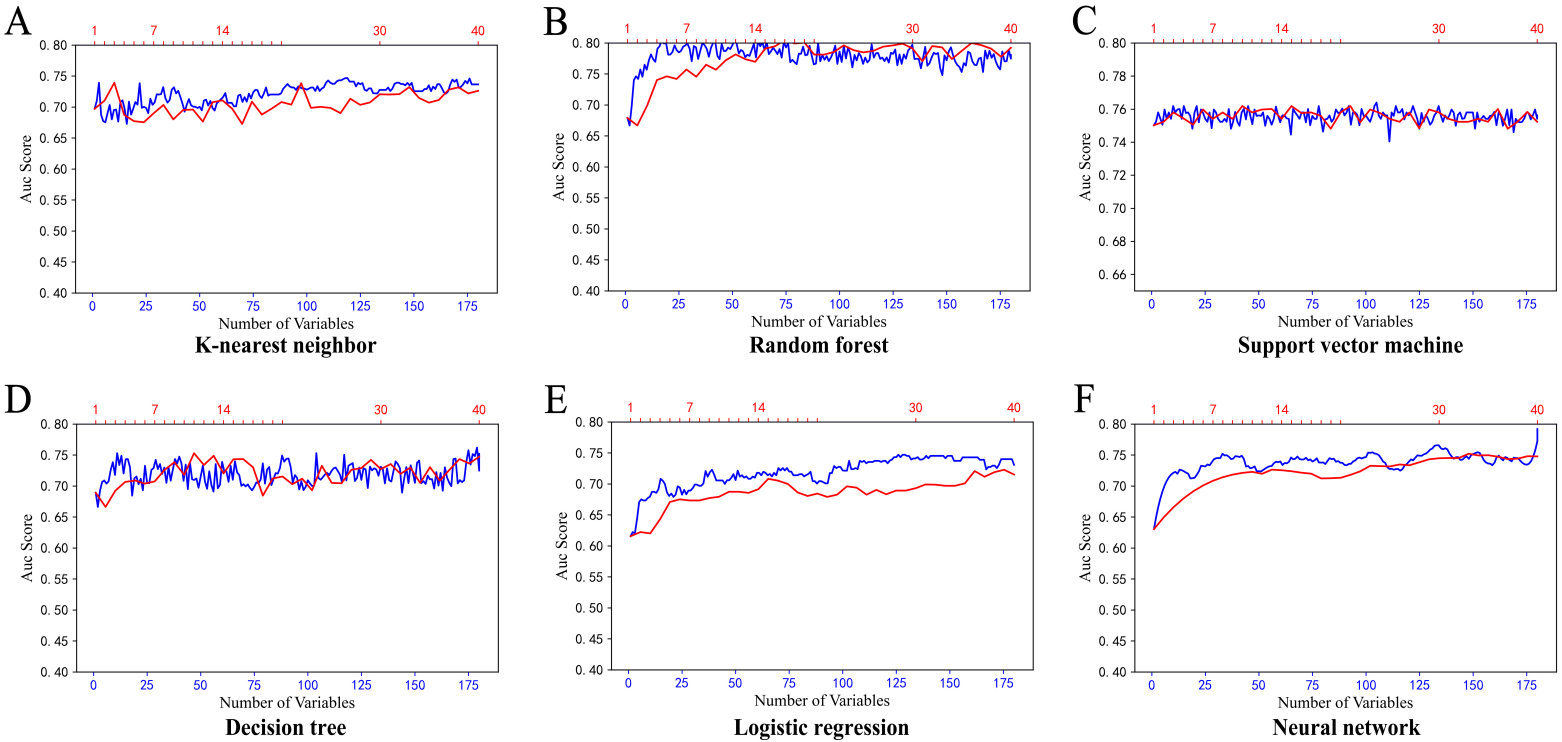
**AUC scores in 6 nested models**. (A) K-nearest neighbor. (B) Random forest. (C) Support vector machine. (D) Decision tree. (E) Logistic regression. And (F) Neural network. Addition of variables caused a change in AUC scores, based on order of variable importance ranking (in blue). The red curve indicates the same but only for the top 40 ranked variables on a magnified scale. Among the six nested models, the random forest classifier reached the highest and changeless AUC score after inputting no less than 20 variables. Introduction of more extra variables to the model had no significant improvement and even an unexpected decline in the AUC score. AUC, area under the curve.

After inputting more than 20 variables, the RF classifier in the six nested models reached a peak AUC score (Fig. 2). Addition of extra variables to the model resulted in no significant improvement but even caused an unexpected decline in the AUC score. Consequently, the top 20 covariates in the RF (top RF-20) were identified, and subsequently enrolled in reconstruction of final models to fetch the reformative classifiers: the K-nearest neighbor with top RF-20, the logistic regression with top RF-20, the decision tree with top RF-20, the RF with top RF-20, the support vector machine with top RF-20, and the neural network with top RF-20.

#### 3.2.2 Performance Evaluation

**Fig. 3.**
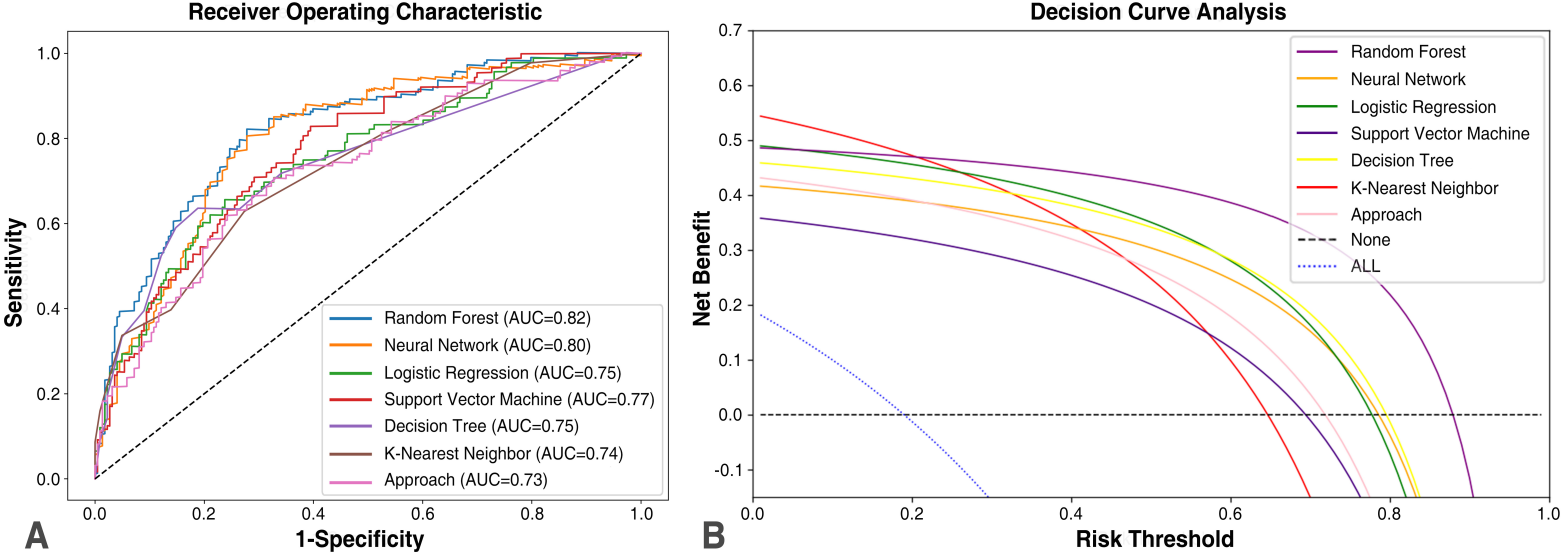
**Model performance of the predictive models**. (A) Receiver operating characteristic curve showing the AUC score for the comparison of predictive model discrimination between patients with and without AKI. (B) Decision curve analysis was performed to evaluate clinical utility. It plots net benefits vs. risk threshold and simulates two scenarios: Treat for none’ and Treat for all’. The analysis reveals that all models conferred clinical benefit over the treat-all and treat-none approach. AUC, area under the curve; AKI, acute kidney injury.

Results from validation of CSA-AKI using an external dataset showed that the six reformative classifiers had high AUCs of the ROC. Notably, the obtained RF had an AUC value of 0.82, which was significantly higher compared with that of others (Fig. 3A). Next, the APPROACH model was recruited to compare results between the traditional logistic regression model and machine learning algorithm. Results showed that the APPROACH model, based on multi-collinearity, had a good performance, although its AUC score (0.73) was still lower than that of other nonlinear models.

To determine the models’ clinical value, DCA curve was generated to quantify the net benefits of predictive models. The DCA delineates the clinical net benefits under several thresholds of probability. Analysis of DCA showed that each model, including APPROACH, yielded net benefits above both extreme lines (“Treat Non” and “Treat All”) in the reasonable threshold range of 0 to 0.9 (Fig. 3B). Notably, RF showed consistently high-level net benefits under the threshold and achieved an efficient execution time for local testing: 54.23 s.

### 3.3 Model Visualization

#### 3.3.1 Tree-Based Prediction

**Fig. 4.**
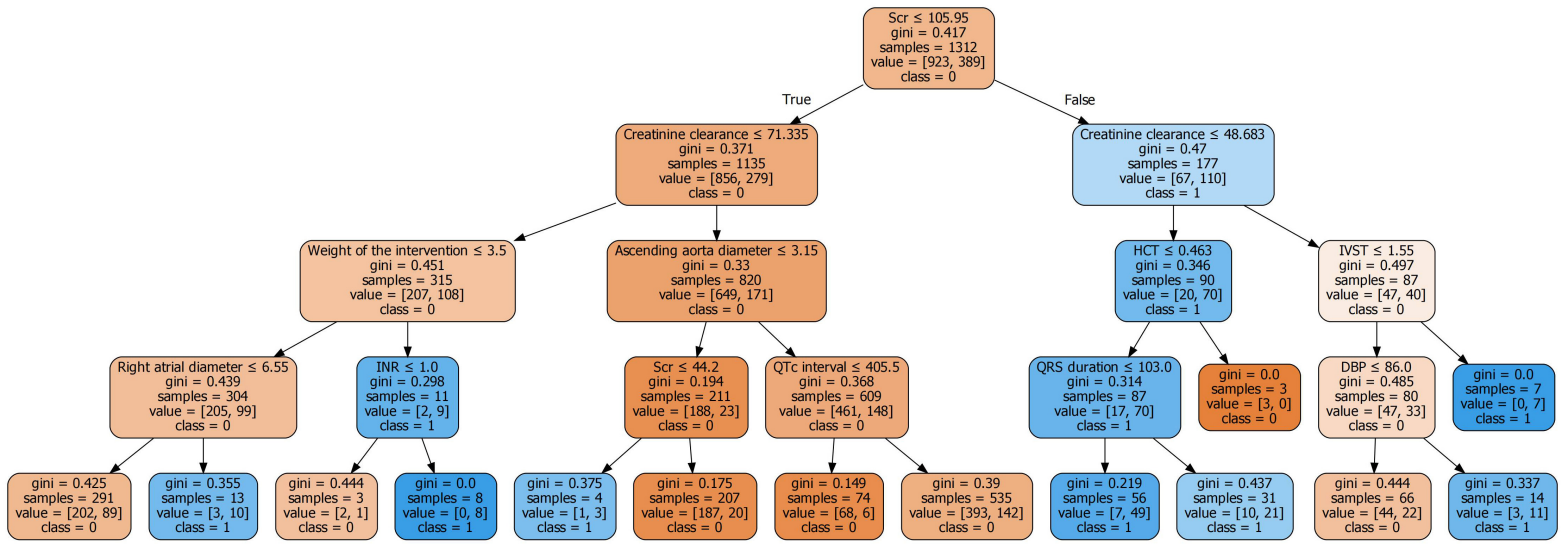
**Binary classification tree plot of patients with (class = 1) and without (class = 0) AKI**. The classification tree starts from a particular profile which best segments the sample, like Scr, and defines the probability of correctly classifying the sample into a classification, i.e., non-AKI or AKI, based on the value of the profile (e.g., 105.95). That division would be assigned with a misclassification probability (i.e., 0.417) and are subsequently delivered to other profile (e.g., creatinine clearance). Color blue and orange indicate the “yes” and “no” classification, respectively. Color densities; color density rises as the Gini index falls. AKI, acute kidney injury; Scr, serum creatinine; HCT, hematocrit; IVST, interventricular septal thickness; INR, international normalized ratio; DBP, diastolic blood pressure.

The common machine learning models founded upon tree-based algorithms included algorithms of decision tree, RF, and support vector machine. A summary of patient stratification, using a tree-like structure, into with (class = yes) and without (class = no) AKI, is presented in Fig. 4.

#### 3.3.2 Feature Importance

**Fig. 5.**
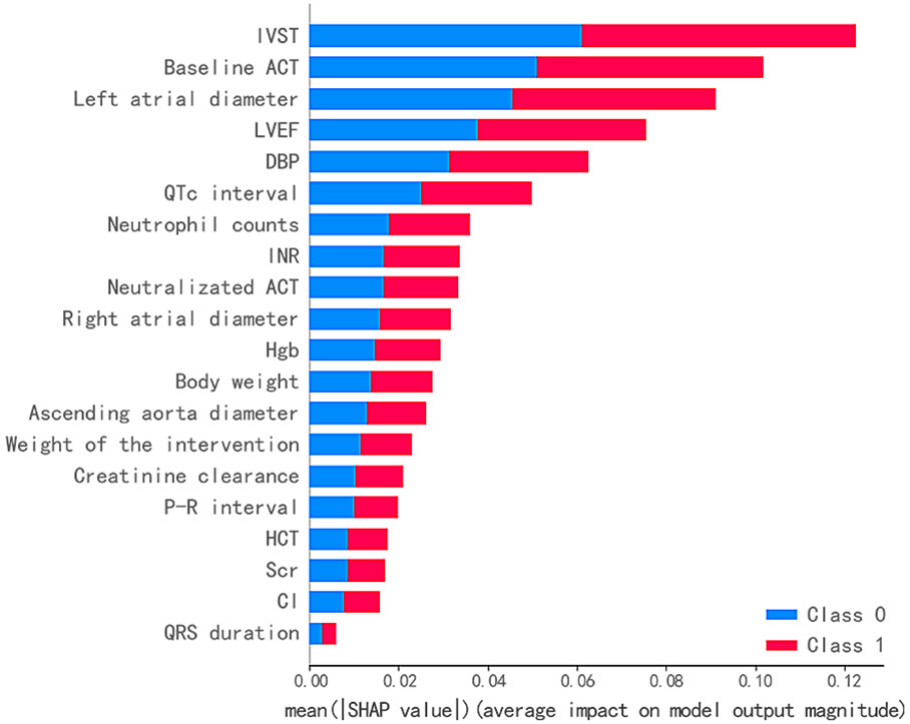
**Feature importance ranking in RF model**. Features on the y-axis are ordered according to their importance, which is defined as the sum of the absolute SHAP values during model development. IVST, interventricular septal thickness; ACT, activated clotting time; LVEF, left ventricular ejection fraction; DBP, diastolic blood pressure; INR, international normalized ratio; Hgb, hemoglobin; HCT, hematocrit; Scr, serum creatinine; Cl, chloride; SHAP, SHapley Additive exPlanation; RF, random forest.

Next, SHAP values from the top RF-20 were computed to determine the feature contribution and interpretability of the predictions in the RF classifier. Summaries of feature importance are illustrated using a density scatter plot and a bar chart (Fig. 5 and **Supplementary Fig. 1**, respectively). Notably, the features were ranked based on descending order of average SHAP values. An overview of SHAP values across all 2108 samples from the variables is shown using a density scatter plot (**Supplementary Fig. 1**). The density plot indicates the number of clustered samples, while the color represents the high (red) and low (blue) values of the feature. Feature values at baseline activated clotting time, left atrial diameter, left ventricular ejection fraction, P-R interval, and serum creatinine were consistent with the SHAP values. A higher feature value for these covariants indicated a greater likelihood of developing CSA-AKI. In contrast, high feature values for hemoglobin, creatinine clearance, and hematocrit implied a low predictive risk of AKI episodes. The averaged impact of the top 20 features on model output is shown in Fig. 5. In summary, the top five variables that significantly contributed to model runs were interventricular septal thickness, baseline activated clotting time, left atrial diameter, left ventricular ejection fraction, and diastolic blood pressure.

#### 3.3.3 Individual Risk Assessment

**Fig. 6.**
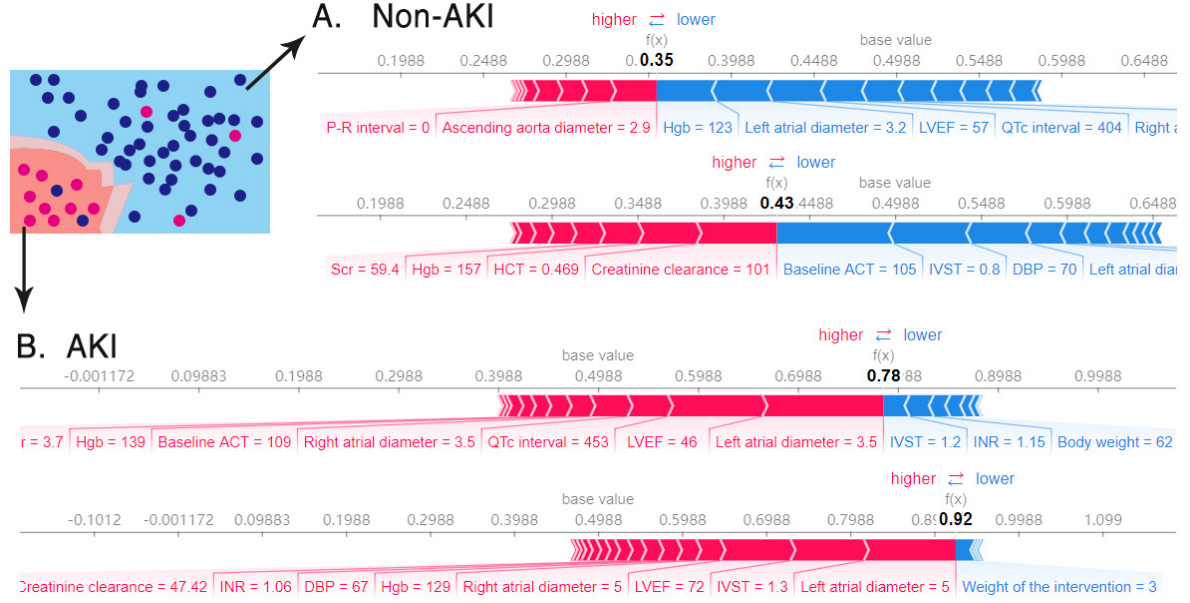
**SHAP feature importance metrics in 4 patients who were correctly stratified into the Non-AKI (A) and AKI (B) groups**. The base value—the mean of the model output (log-odds) over the training dataset—was 0.4988. Output values (bold), expressed as log odds ratio of probability of AKI to probability of Non-AKI (i.e., log ($\frac{P\,\left(a\,k\,i\right)}{1-P\,\left(a\,k\,i\right)}$)), that are low (0.35, 0.43) in Non-AKI patients (A) and high (0.78, 0.92) in AKI patients. (B) Red and blue bars indicate increasing and decreasing probability of AKI, respectively. The size of the bars depicts each feature’s contribution to the model’s output. AKI, acute kidney injury; Hgb, hemoglobin; LVEF, left ventricular ejection fraction; Scr, serum creatinine; HCT, hematocrit; ACT, activated clotting time; IVST, interventricular septal thickness; DBP, diastolic blood pressure; INR, international normalized ratio.

Single prediction at the individual level was visualized in terms of accountable outputs with and without predictive AKI outcomes (Fig. 6 and **Supplementary Fig. 2**). The SHAP importance metrics identified 8 patients who were correctly (Fig. 6) or incorrectly (**Supplementary Fig. 2**) predicted to develop AKI or not, which increased “black box” disclosure and resulting in clinically interpretable results. In Fig. 6A, contributors like hemoglobin and left atrial diameter were considered to be the most important protectors against AKI, while in Fig. 6B, contributors like left atrial diameter and LVEF were considered to be the most important promotors of AKI.

#### 3.3.4 Feature-Driven Prediction

**Fig. 7.**
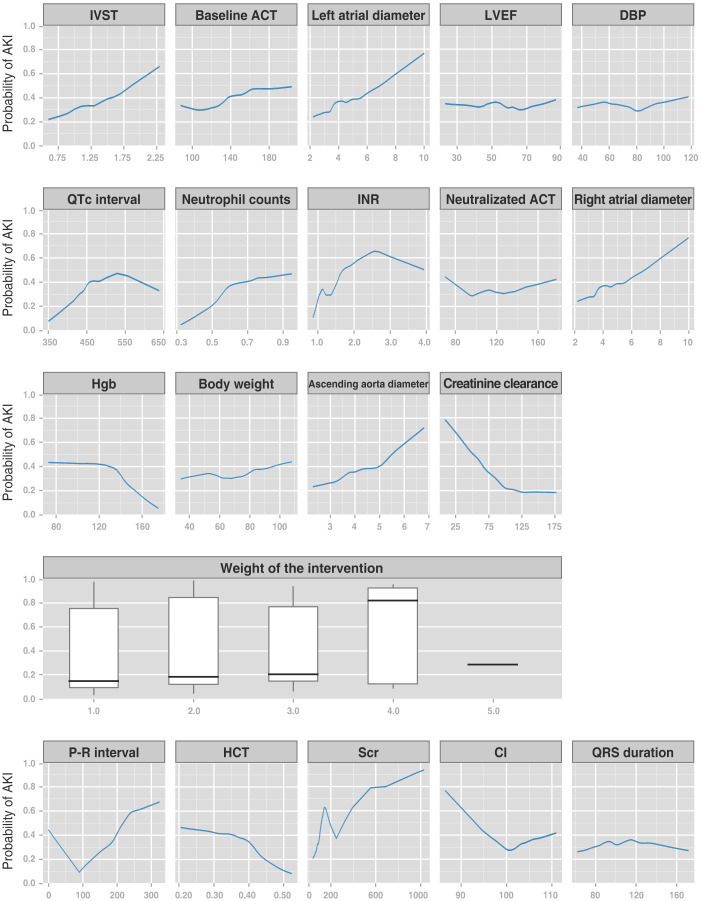
**Plots showing the Lowess curves (for continuous variables) and box plots (for categorical variables) of the AKI probability vs. variable values for the top RF-20 predictors**. The y-axis represents predictive probability calculated from the RF-20 algorithm (range: 0 to 1). The x-axis spans the range (or categories) of the top RF-20 predictors. IVST, interventricular septal thickness; ACT, activated clotting time; LVEF, left ventricular ejection fraction; DBP, diastolic blood pressure; INR, international normalized ratio; Hgb, hemoglobin; HCT, hematocrit; Scr, serum creatinine; Cl, chloride; AKI, acute kidney injury; RF, random forest.

The relationship among variables and outcomes is presented using a heatmap (**Supplementary Fig. 3**). Regarding interest outcomes, AKI exhibited weak positive correlations with the levels of baseline activated clotting time, left atrial diameter, and serum creatinine (slight blue in the heatmap). On the other hand, AKI had a weak negative correlation with levels of hemoglobin, creatinine clearance, and hematocrit (slight yellow in the heatmap). Collectively, these results did not reveal any evidence of leading factors but the existence of a multifactorial involvement process during AKI development. Furthermore, the feature tendency plot was constructed to improve our understanding of the effects of a single variable on the predictive outcomes during modeling (Fig. 7). The generated plots, comprising curves (for continuous variables) and box plots (for categorical variables) of the AKI probability vs. variable values for the top RF-20 predictors, revealed the changing contribution of each variable as its different values were taken.

## 4. Discussion

In this study, we aimed to build machine learning methods for predicting patients with a high probability of developing acute kidney injury after cardiac surgery (Fig. 6). The results demonstrated that machine learning algorithms offer great potential for risk stratification of AKI episodes in patients after cardiac surgery. Initially, the nested models were assembled based on six machine learning algorithms using 126 preoperative and perioperative variables, and the top 20 predictors in the RF classifier were selected to reconstruct prediction models. This work is unique because it demonstrates the patterns of variables that vary for specific AUC scores to identify a suitable algorithm and variable selection. Moreover, this study differs in several ways from previous studies: (1) This was a multicenter study with external validation of the models, and notably, the predictive performance of the models was compared to that of an existing risk model. (2) The DCA curve provided an external assessment of the clinical benefits of the model in clinical practice. (3) Interpretable machine learning was performed, which not only showed individual-level predictive evidence for the patients but also identified some predictors that were not “ignored” by traditional logistic regression analyses.

The RF classifier with the top 20 predictors showed the best performance in deep phenotyping an independent patient cohort, as evidenced by an AUC score of 0.82. As far as we know, the original empirical research describing CSA-AKI using a machine learning algorithm reported that the highest AUC was achieved in a gradient boosting machine [15]. Specifically, the study comprehensively compared machine learning and traditional logistic regression methods, as well as previously reported risk models. Machine learning methods had the highest performance outcome, as evidenced by an AUC value of 0.78, while the risk score models had poor performance (AUC ranging from 0.55 to 0.58), which can be attributed to the fact that the number of included candidates is poor and perioperative parameters were missing [15]. Jiang *et al*. [21] reviewed the efficiency of previously established models, based on orthodox multilinear regression methods, in predicting AKI after cardiac surgery. They obtained AUC values ranging from 0.60 to 0.70. In the present study, the APPROACH risk model generated an AUC score of 0.73, indicating that the RF-based method had a better prediction outcome than traditional risk score. This phenomenon could be partially attributed to the fact that the machine learning techniques employ superior algorithms when dealing with overfitting and nonlinearities. To investigate the potential clinical benefit of our predictive model, the DCA, a statistical method for estimating the clinical impact of using the predictive model, was performed [22]. This DCA provided complementary information which can help the decision-making process. Our analyses showed that in contrast to the case that no predictive models were available, the RF classifier yielded beneficial clinical benefits along a 0–90% decision threshold.

Furthermore, the SHAP plot displayed the most influential top-20 predictors based on the RF classifier for model prediction. These results revealed similar predictors to those obtained in previous machine learning applications in CSA-AKI, such as hemoglobin, serum creatinine, left ventricular ejection fraction, and hematocrit [15, 19]. However, some discrepancies may arise, partly due to differences in sample sources for the dataset as well as disparate algorithms employed during modeling. Among the top predictors of disease outcomes, several traditional risk factors known to be associated with AKI episodes were also represented. Nevertheless, it should be noted that the lower-than-expected performance of traditional risk factors observed herein might arise from the fact that these variables are triggers or fundamental elements of other subclinical factors, especially sub-phenotypes which are not terminal to disease inception but are closely associated with development of adverse outcomes. Regardless, some of them are still critical in clinical practice, especially in disease prevention.

The top RF-20 variables identified in this study represent novel predictors that are not typically incorporated in established risk scores for the detection of AKI development. These include coagulation indicators (activated clotting time and international normalized ratio), echocardiography findings (ascending aorta diameter, left/right atrial diameter, and interventricular septal thickness), and electrocardiography feature (P-R interval, QTc interval, and QRS duration). However, little is known regarding echocardiographic and electrocardiographic interpretation on the one hand and diagnostic possibilities in clinical practice with regard to AKI on the other hand. Generally, large portions of electrocardiographic signals are frequently ignored by many clinicians. However, QRS widening in electrocardiography may be a manifestation of left ventricular structure abnormalities (e.g., left ventricular mass or increased dimension) but may also indicate a malfunction, such as left ventricular systolic dysfunction. A study conducted by Ilkhanoff *et al*. [23] in 2012, containing 4591 people with a mean follow-up of 7.1 years, found that QRS >100 ms was significantly associated with magnetic resonance imaging (MRI) measures of cardiac structure and function, as well as heart failure events. This implies that the QRS duration may be a useful marker of left ventricular function [23]. In addition, left atrial diameter from echocardiography was found to be negatively associated with left ventricular ejection fraction while positively with N-terminal pro B-type natriuretic peptide (NT-proBNP) [24, 25]. Previous data of left atrial size concerning renal outcomes also indicate that the left atrial diameter can be used for predicting the risk of adverse renal outcomes [26]. Nonetheless, in most clinical cases, not all information obtained is used for AKI diagnosis. But results of the present study offer promise that machine learning is a robust alternative approach to provide powerful interpretation and utilization of results obtained from established screening tools, such as electrocardiography and echocardiography. Moreover, although interventricular septal thickness and baseline activated clotting time dominated the predictive outcomes of AKI in all samples, other predictors like ascending aorta diameter and left atrial diameter also played a central role in individual sample prediction. This is expected to guide future research works seeking to discover new predictors.

Analysis of the role of individual features within the RF model revealed that the prediction performance was not attributed to a single leading predictor. Instead, the full context of the selected predictors plays a crucial role in the observed outcomes. Furthermore, the influence of certain predictors on model outcome was identified, especially the heretofore underestimated factors such as interventricular septal thickness and activated clotting time, or unknown biological relationships, which are emerging at the forefront due to machine learning. Collectively, our results indicate that machine learning techniques open the possibilities of proactively investigating unidentified relationships or extracting new useful biomarkers, which is beneficial for understanding disease pathogenesis and appointing new path toward intervention [27, 28]. Considering the fact that the world is moving into the era of personalized medicine and big data, machine learning presents novel frameworks and new approaches for data analysis in a way that is beyond the capacity of traditional statistical approaches [29, 30].

Results from a previous study demonstrated that early initiation of renal replacement therapy significantly reduced 90-day mortality rates in patients with hospital-acquired AKI patients relative to those who had delayed initiation of renal replacement therapy [31]. Notably, early intervention heavily relies on close monitoring, early detection, execution of kidney prophylaxes, and prevention of inaccurate diagnoses. However, previously established models failed to achieve early detection in clinical practice due to their modest external performance. Therefore, leveraging machine learning models into novel intelligent decision-support systems might contribute to the effective stratification of patients at a high risk of CSA-AKI, even before the serum creatinine changes. In the future, incorporating such risk-stratified care management into an early warning health management system opens the possibility of prospectively identifying patients at high risk, thus allowing accurate assessment of patient’s situation and offering them care management services for preventive care [32].

Nevertheless, this study has some limitations. Firstly, the observed effects may differ in a greater data set with differently distributed sample profiles. Secondly, the small number of participants compromises the generalizability of our discovery, and its retrospective nature limits our ability to make any causal determinations. Notably, although feature selection by nested modeling can minimize the noise of overfitting, some potentially valuable covariates might be spared in this process. In addition, the urine criteria in Kidney Disease Improving Global Outcomes (KDIGO) were exempted in defining AKI, which may yield a negative impact on the results. Currently, we only applied machine learning to all stage CSA-AKI but not to higher stages of CSA-AKI (stages 2–3), an area that will be the subject of future investigations. While we showed a possibility of machine learning-guided risk stratification, it is not clear whether the results can be translated to improve the clinical management of patients [33], necessitating further prospective investigations.

## 5. Conclusions

This study demonstrates that machine learning techniques can be successfully applied to screen out individuals at high risk of developing postoperative AKI among patients who undergo cardiac surgery. Given that AKI is associated with high morbidity and mortality rates among hospitalized patients, especially those undergoing cardiac surgery, the data-driven, AI-based risk estimator may be of great clinical benefit to guide clinicians during clarification of the underlying complex relationships of disease pathogenesis as well as identification of individuals with a likelihood of developing CSA-AKI. However, further studies are required to improve the classification accuracy of the estimator by combining AKI biomarkers, like neutrophil gelatinase-associated lipocalin (NGAL), kidney injury molecule 1 (KIM-1), and interleukin 18 (IL-18). Future work should assess the clinical effectiveness and cost-effectiveness of the model under a real-world context, as well as the impact of decision-making on delivering treatment strategy.

## Data Availability

The datasets used and/or analyzed during the current study are available from the corresponding author on reasonable request.

## Supplementary Material

Supplementary material associated with this article can be found, in the online version, at https://doi.org/10.31083/j.rcm2408229.
