# Supplementary material for: Development, External Validation, and Visualization of Machine Learning Models for Predicting Occurrence of Acute Kidney Injury after Cardiac Surgery
Source: Rev Cardiovasc Med. 2023 Aug 9;24(8):229. doi: 10.31083/j.rcm2408229 (PMC11266781; doi:10.31083/j.rcm2408229)
Supplement: Supplementary file 1 [file 2153-8174-24-8-229-s1.docx]

Supplementary Table 1. Perioperative characteristics of patients developed acute kidney injury or not after cardiac surgery

| **Characteristic** | **Overall** | **Non-AKI** | **AKI** | **p-Value** |
| --- | --- | --- | --- | --- |
| **Patient population, n** | **2108** | **1471** | **637** |  |
| **Demographic data** |  |  |  |  |
| Male, n (%) | 1323 (62.76%) | 916 (62.27%) | 407 (63.89%) | 0.510 |
| Age, median (IQR) (y) | 59.00 (50.00, 66.00) | 57.00 (48.00, 65.00) | 62.00 (54.00, 69.00) | < 0.001^*^ |
| Height, median (IQR) (m) | 165.00 (158.00, 171.00) | 165.00 (158.00, 171.00) | 165.00 (158.00, 170.00) | 0.859 |
| Weight, median (IQR) (kg) | 67.00 (59.00, 76.00) | 67.00 (59.00, 75.65) | 68.00 (59.00, 78.00) | 0.225 |
| BMI, median (IQR) (kg/m^2^) | 24.96 (22.37, 27.29) | 24.91 (22.32, 27.10) | 25.03 (22.41, 27.55) | 0.186 |
| SBP, median (IQR) (mmHg) | 127.00 (114.00, 141.00) | 125.00 (112.00, 140.00) | 131.00 (116.00, 146.00) | < 0.001^*^ |
| DBP, median (IQR) (mmHg) | 72.00 (64.00, 80.00) | 72.00 (64.00, 80.00) | 73.00 (63.00, 81.00) | 0.496 |
| MAP, median (IQR) (mmHg) | 90.00 (82.33, 99.67) | 89.67 (82.33, 98.67) | 91.33 (82.67, 101) | 0.004^*^ |
| Smoking, n (%) | 768 (36.48%) | 521 (35.47%) | 247 (38.84%) | 0.154 |
| Alcohol, n (%) | 404 (19.19%) | 269 (18.31%) | 135 (21.23%) | 0.134 |
| EUROSCORE II Score, median (IQR) | 1.83 (0.93, 3.47) | 1.53 (0.84, 2.88) | 2.76 (1.31, 5.65) | < 0.001^*^ |
| CCS class 4, n (%) | 90 (4.27%) | 46 (3.13%) | 44 (6.91%) | < 0.001^*^ |
| NYHA Functional Classification, n (%) |  |  |  |  |
| 1 | 490 (23.24%) | 353 (24.00%) | 137 (21.51%) | < 0.001^*^ |
| 2 | 819 (38.85%) | 597 (40.58%) | 222 (34.85%) |  |
| 3 | 680 (32.26%) | 457 (31.07%) | 223 (35.01%) |  |
| 4 | 119 (5.65%) | 64 (4.35%) | 55 (8.63%) |  |
| ASA Physical Status Classification, n (%) |  |  |  |  |
| 1 | 10 (0.48%) | 8 (0.55%) | 2 (0.32%) | < 0.001^*^ |
| 2 | 335 (16.15%) | 269 (18.55%) | 66 (10.58%) |  |
| 3 | 1114 (53.71%) | 785 (54.14%) | 329 (52.72%) |  |
| 4 | 613 (29.56%) | 388 (26.76%) | 225 (36.06%) |  |
| 5 | 2 (0.10%) | 0 (0.00%) | 2 (0.32%) |  |
| Mallampati Airway Classification, n (%) |  |  |  |  |
| 1 | 868 (41.95%) | 632 (43.65%) | 236 (38.00%) | 0.036^*^ |
| 2 | 546 (26.39%) | 383 (26.45%) | 163 (26.25%) |  |
| 3 | 633 (30.59%) | 420 (29.01%) | 213 (34.30%) |  |
| 4 | 22 (1.06%) | 13 (0.90%) | 9 (1.45%) |  |
| **Medical history** |  |  |  |  |
| Dyslipidemia, n (%) | 159 (7.54%) | 98 (6.66%) | 61 (9.58%) | 0.025^*^ |
| Diabetes mellitus, n (%) | 397 (18.83%) | 241 (16.38%) | 156 (24.49%) | < 0.001^*^ |
| Diabetes on insulin, n (%) | 214 (10.15%) | 121 (8.22%) | 93 (14.60%) | < 0.001^*^ |
| Hypertension, n (%) | 922 (43.74%) | 562 (38.21%) | 360 (56.51%) | < 0.001^*^ |
| Chronic kidney disease, n (%) | 74 (3.51%) | 19 (1.29%) | 55 (8.63%) | < 0.001^*^ |
| Infectious endocarditis, n (%) | 93 (4.41%) | 68 (4.62%) | 25 (3.92%) | 0.548 |
| Neurological dysfunction, n (%) | 126 (5.98%) | 83 (5.64%) | 43 (6.75%) | 0.376 |
| Pulmonary hypertension, n (%) |  |  |  |  |
| Mild | 447 (21.20%) | 314 (21.35%) | 133 (20.88%) | 0.042^*^ |
| Moderate | 219 (10.39%) | 137 (9.31%) | 82 (12.87%) |  |
| Severe | 62 (2.94%) | 49 (3.33%) | 13 (2.04%) |  |
| **Preoperative condition** |  |  |  |  |
| MI within 90 days, n (%) | 93 (4.41%) | 55 (3.74%) | 38 (5.97%) | 0.030^*^ |
| Critical preoperative state, n (%) | 193 (9.16%) | 90 (6.12%) | 103 (16.17%) | < 0.001^*^ |
| Previous cardiac surgery, n (%) | 343 (16.27%) | 219 (14.89%) | 124 (19.47%) | 0.011^*^ |
| Number of previous cardiac operations, n (%) |  |  |  |  |
| 1 | 328 (15.56%) | 209 (14.21%) | 119 (18.68%) | 0.043^*^ |
| 2 | 10 (0.47%) | 6 (0.41%) | 4 (0.63%) |  |
| 3 | 5 (0.24%) | 4 (0.27%) | 1 (0.16%) |  |
| Preoperative coronary angiography, n (%) | 1370 (64.99%) | 938 (63.77%) | 432 (67.82%) | 0.082 |
| Preoperative RRT, n (%) | 15 (0.71%) | 3 (0.20%) | 12 (1.88%) | < 0.001^*^ |
| Atrial flutter, n (%) | 47 (2.23%) | 29 (1.97%) | 18 (2.83%) | 0.289 |
| Atrial fibrillation, n (%) | 452 (21.44%) | 278 (18.90%) | 174 (27.32%) | < 0.001^*^ |
| **Preoperative medications** |  |  |  |  |
| Digoxin, n (%) | 715 (33.92%) | 499 (33.92%) | 216 (33.91%) | 1.000 |
| β-block, n (%) | 1165 (55.27%) | 760 (51.67%) | 405 (63.58%) | < 0.001^*^ |
| ACEi, n (%) | 200 (9.49%) | 126 (8.57%) | 74 (11.62%) | 0.034^*^ |
| ARB, n (%) | 259 (12.29%) | 162 (11.01%) | 97 (15.23%) | 0.008^*^ |
| Calcium channel blockers, n (%) | 737 (34.96%) | 452 (30.73%) | 285 (44.74%) | < 0.001^*^ |
| Diuretics, n (%) | 1566 (74.29%) | 1078 (73.28%) | 488 (76.61%) | 0.121 |
| Anticoagulants, n (%) | 617 (29.27%) | 378 (25.70%) | 239 (37.52%) | < 0.001^*^ |
| Aspirin, n (%) | 443 (21.02%) | 296 (20.12%) | 147 (23.08%) | 0.141 |
| Statins, n (%) | 595 (28.23%) | 394 (26.78%) | 201 (31.55%) | 0.029^*^ |
| Insulin, n (%) | 224 (10.63%) | 128 (8.70%) | 96 (15.07%) | < 0.001^*^ |
| OHA, n (%) | 307 (14.56%) | 198 (13.46%) | 109 (17.11%) | 0.034^*^ |
| **Electrocardiographic detail** |  |  |  |  |
| PR interval, median (IQR) (ms) | 150.00 (112.00, 172.00) | 152.00 (124.00, 171.00) | 148.00 (0.00, 172.00) | 0.084 |
| QRS duration, median (IQR) (ms) | 96.00 (86.00, 106.00) | 96.00 (86.00, 106.00) | 96.00 (86.00, 108.00) | 0.236 |
| QT interval, median (IQR) (ms) | 392.00 (364.00, 418.00) | 392.00 (364.00, 418.00) | 394.00 (368.00, 422.00) | 0.158 |
| QTc interval, median (IQR) (ms) | 439.00 (419.00, 457.00) | 435.00 (417.00, 454.00) | 444.00 (424.00, 462.00) | < 0.001^*^ |
| Abnormal T wave, n (%) | 1322 (62.90%) | 869 (59.20%) | 453 (71.56%) | < 0.001^*^ |
| Abnormal Q wave, n (%) | 231 (10.99%) | 156 (10.63%) | 75 (11.85%) | 0.456 |
| **Echocardiographic detail** |  |  |  |  |
| Ascending aorta diameter, median (IQR) (cm) | 3.50 (3.20, 4.00) | 3.50 (3.10, 3.90) | 3.60 (3.30, 4.10) | < 0.001^*^ |
| Left atrial diameter, median (IQR) (cm) | 4.00 (3.50, 4.90) | 4.00 (3.50, 4.80) | 4.20 (3.60, 5.00) | < 0.001^*^ |
| LVEDD, median (IQR) (cm) | 4.80 (4.20, 5.50) | 4.80 (4.20, 5.50) | 4.80 (4.20, 5.50) | 0.299 |
| IVST, median (IQR) (cm) | 1.10 (1.00, 1.20) | 1.10 (1.00, 1.20) | 1.10 (1.00, 1.30) | < 0.001^*^ |
| LVPWT, median (IQR) (cm) | 1.10 (1.00, 1.20) | 1.10 (0.90, 1.20) | 1.10 (1.00, 1.30) | < 0.001^*^ |
| Right atrial diameter, median (IQR) (cm) | 3.60 (3.20, 4.10) | 3.50 (3.20, 4.00) | 3.60 (3.20, 4.30) | 0.003^*^ |
| RVD, median (IQR) (cm) | 3.00 (2.70, 3.40) | 3.00 (2.70, 3.40) | 3.10 (2.70, 3.50) | 0.256 |
| MPAD, median (IQR) (cm) | 2.50 (2.30, 2.80) | 2.50 (2.30, 2.77) | 2.60 (2.30, 2.80) | < 0.001^*^ |
| LVEF, median (IQR) (%) | 61.00 (55.00, 67.00) | 61.00 (55.00, 68.00) | 60.00 (53.00, 67.00) | 0.004^*^ |
| mPAP, median (IQR) (mmHg) | 22.00 (22.00, 41.00) | 22.00 (22.00, 40.00) | 22.00 (22.00, 46.00) | 0.345 |
| **Laboratory findings** |  |  |  |  |
| Hgb, median (IQR) (g/L) | 134.00 (119.00, 146.50) | 136.00 (122.00, 148.00) | 127.00 (111.00, 140.00) | < 0.001^*^ |
| RBC, median (IQR) (10^12^/L) | 4.36 (3.96, 4.79) | 4.43 (4.07, 4.86) | 4.18 (3.69, 4.62) | < 0.001^*^ |
| WBC, median (IQR) (10^9^/L) | 6.20 (5.13, 7.48) | 6.14 (5.12, 7.33) | 6.33 (5.14, 7.94) | 0.022^*^ |
| Neutrophil percentage, median (IQR) (%) | 0.61 (0.54, 0.68) | 0.59 (0.53, 0.66) | 0.63 (0.57, 0.71) | < 0.001^*^ |
| Lymphocyte percentage, median (IQR) (%) | 0.29 (0.22, 0.35) | 0.30 (0.24, 0.36) | 0.25 (0.19, 0.32) | < 0.001^*^ |
| PLT, median (IQR) (10^9^/L) | 191.25 (154.00, 235.00) | 196.00 (159.00, 236.00) | 179.00 (140.00, 228.00) | < 0.001^*^ |
| HCT, median (IQR) (L/L) | 0.39 (0.351, 0.42) | 0.40 (0.36, 0.43) | 0.37 (0.33, 0.41) | < 0.001^*^ |
| APTT, median (IQR) (s) | 37.10 (34.30, 40.50) | 36.75 (34.00, 40.00) | 37.75 (34.80, 41.82) | < 0.001^*^ |
| INR, median (IQR) | 1.06 (1.00, 1.15) | 1.05 (1.00, 1.13) | 1.08 (1.01, 1.19) | < 0.001^*^ |
| Plasma fibrinogen, median (IQR) (g/L) | 3.10 (2.60, 3.75) | 3.01 (2.57, 3.65) | 3.31 (2.69, 3.98) | < 0.001^*^ |
| ALT, median (IQR) (U/L) | 18.00 (12.40, 27.70) | 18.70 (12.70, 28.30) | 16.60 (11.40, 26.30) | 0.001^*^ |
| AST, median (IQR) (U/L) | 18.20 (14.70, 24.40) | 18.20 (14.90, 23.90) | 18.30 (14.40, 25.80) | 0.672 |
| Total protein, median (IQR) (g/L) | 67.50 (63.60, 71.60) | 67.80 (64.10, 71.70) | 66.95 (62.60, 71.40) | 0.001^*^ |
| Albumin, median (IQR) (g/L) | 40.80 (38.00, 43.30) | 41.20 (38.70, 43.70) | 39.50 (36.60, 42.34) | < 0.001^*^ |
| Total bilirubin, median (IQR) (umol/L) | 12.60 (9.30, 17.80) | 12.63 (9.40, 17.75) | 12.50 (8.83, 18.00) | 0.690 |
| Direct bilirubin, median (IQR) (umol/L) | 4.10 (2.80, 6.00) | 4.00 (2.80, 5.80) | 4.27 (2.80, 6.70) | 0.037^*^ |
| Blood glucose, median (IQR) (mmol/L) | 5.08 (4.59, 6.05) | 5.01 (4.56, 5.83) | 5.28 (4.70, 6.52) | < 0.001^*^ |
| Scr, median (IQR) (umol/L) | 79.05 (67.6, 91.819) | 76.7 (66.27, 87.8) | 85.6 (72.1, 106) | < 0.001^*^ |
| Creatinine clearance, median (IQR) (ml/min) | 80.85 (64.46, 99.65) | 84.73 (69.49, 102.66) | 71.43 (53.73, 89.48) | < 0.001^*^ |
| Urea nitrogen, median (IQR) (mmol/L) | 5.88 (4.75, 7.44) | 5.62 (4.55, 6.95) | 6.69 (5.26, 9.15) | < 0.001^*^ |
| Serum potassium, median (IQR) (mmol/L) | 4.02 (3.77, 4.28) | 4.01 (3.76, 4.25) | 4.07 (3.79, 4.37) | < 0.001^*^ |
| Serum sodium, median (IQR) (mmol/L) | 141.20 (139.44, 142.90) | 141.40 (139.70, 143.00) | 140.87 (138.90, 142.60) | < 0.001^*^ |
| Cl, median (IQR) (mmol/L) | 102.70 (100.25, 104.90) | 102.70 (100.40, 104.80) | 102.70 (99.90, 105.20) | 0.970 |
| **Type of surgery, n (%)** |  |  |  |  |
| Valve surgery only | 1160 (55.03%) | 852 (57.92%) | 308 (48.35%) | < 0.001^*^ |
| CABG only | 595 (28.23%) | 432 (29.37%) | 163 (25.59%) |  |
| CABG+Valve | 171 (8.11%) | 87 (5.91%) | 84 (13.19%) |  |
| Surgery on thoracic aorta | 182 (8.63%) | 100 (6.80%) | 82 (12.87%) |  |
| Minimally invasive, n (%) | 386 (18.31%) | 298 (20.26%) | 88 (13.81%) | < 0.001^*^ |
| Emergency, n (%) | 105 (4.98%) | 38 (2.58%) | 67 (10.52%) | < 0.001^*^ |
| Weight of the intervention^1^, n (%) |  |  |  |  |
| 1 | 1218 (57.78%) | 891 (60.57%) | 327 (51.33%) | < 0.001^*^ |
| 2 | 516 (24.48%) | 340 (23.11%) | 176 (27.63%) |  |
| 3 | 312 (14.80%) | 208 (14.14%) | 104 (16.33%) |  |
| 4 | 58 (2.75%) | 29 (1.97%) | 29 (4.55%) |  |
| 5 | 4 (0.19%) | 3 (0.20%) | 1 (0.16%) |  |
| **Intraoperative variables** |  |  |  |  |
| Anesthesia time, median (IQR) (h) | 5.58 (4.75, 6.67) | 5.50 (4.67, 6.33) | 6.00 (5.00, 7.50) | < 0.001^*^ |
| Operation time, median (IQR) (h) | 4.72 (4.00, 5.77) | 4.58 (3.92, 5.50) | 5.17 (4.17, 6.58) | < 0.001^*^ |
| CPB time, median (IQR) (min) | 125.00 (91.00, 171.00) | 120.00 (88.00, 158.00) | 138.00 (98.00, 199.00) | < 0.001^*^ |
| Cross clamp time, median (IQR) (min) | 92.00 (65.00, 127.00) | 89.00 (64.00, 123.00) | 98.00 (68.00, 139.00) | < 0.001^*^ |
| IV-pumped nitroglycerin, n (%) | 865 (41.03%) | 567 (38.55%) | 298 (46.78%) | < 0.001^*^ |
| Epinephrine administration, n (%) | 291 (13.80%) | 126 (8.57%) | 165 (25.90%) | < 0.001^*^ |
| Norepinephrine administration, n (%) | 27 (1.28%) | 7 (0.48%) | 20 (3.14%) | < 0.001^*^ |
| Amiodarone administration, n (%) | 228 (10.82%) | 136 (9.25%) | 92 (14.44%) | < 0.001^*^ |
| Cryoprecipitate treatment, n (%) | 119 (5.65%) | 61 (4.15%) | 58 (9.11%) | < 0.001^*^ |
| Novoseven therapy, n (%) | 90 (4.27%) | 33 (2.24%) | 57 (8.95%) | < 0.001^*^ |
| Defibrillation treatment, n (%) | 714 (33.87%) | 495 (33.65%) | 219 (34.38%) | 0.783 |
| Number of defibrillation attempt, n (%) |  |  |  |  |
| 0 | 1394 (66.13%) | 976 (66.35%) | 418 (65.62%) | 0.684 |
| 1 | 603 (28.61%) | 423 (28.76%) | 180 (28.26%) |  |
| 2 | 81 (3.84%) | 54 (3.67%) | 27 (4.24%) |  |
| 3 | 25 (1.19%) | 16 (1.09%) | 9 (1.41%) |  |
| 4 | 3 (0.14%) | 1 (0.07%) | 2 (0.31%) |  |
| 5 | 2 (0.09%) | 1 (0.07%) | 1 (0.16%) |  |
| Temporary pacemaker implantation, n (%) | 296 (14.40%) | 189 (13.16%) | 107 (17.26%) | 0.018^*^ |
| Perioperative blood loss, median (IQR) (ml) | 400.00 (300.00, 500.00) | 400.00 (300.00, 400.00) | 400.00 (300.00, 600.00) | < 0.001^*^ |
| Perioperative blood loss, median (IQR) (ml/kg) | 5.57 (4.17, 7.49) | 5.33 (3.95, 6.94) | 6.54 (4.76, 9.23) | < 0.001^*^ |
| Perioperative blood loss, median (IQR) (ml/kg/h) | 1.17 (0.88, 1.56) | 1.13 (0.84, 1.51) | 1.28 (0.97, 1.65) | < 0.001^*^ |
| Urine output, median (IQR) (ml) | 860.0 (470.0, 1350.0) | 950.0 (550.0, 1400.0) | 640.0 (300.0, 1100.0) | < 0.001^*^ |
| Urine output, median (IQR) (ml/kg) | 12.79 (7.02, 20.52) | 14.17 (8.42, 21.75) | 9.82 (4.23, 16.92) | < 0.001^*^ |
| Urine output, median (IQR) (ml/kg/h) | 2.68 (1.49, 4.18) | 3.07 (1.87, 4.49) | 1.82 (0.81, 3.24) | < 0.001^*^ |
| Dosage of plasma substitute, median (IQR) (ml) | 0.00 (0.00, 0.00) | 0.00 (0.00, 0.00) | 0.00 (0.00, 500.00) | 0.003^*^ |
| 20% Albumin, median (IQR) (ml) | 100.00 (100.00, 100.00) | 100.00 (100.00, 100.00) | 100.00 (100.00, 150.00) | < 0.001^*^ |
| Ringer lactate solution, median (IQR) (ml) | 1400.0 (1300.0, 1400.0) | 1400.0 (1300.0, 1400.0) | 1400.0 (1300.0, 1400.0) | 0.285 |
| 5%NaHCO3, median (IQR) (ml) | 300.00 (250.00, 400.00) | 300.00 (250.00, 350.00) | 350.00 (250.00, 500.00) | < 0.001^*^ |
| 25% Mannitol, median (IQR) (ml) | 200.00 (150.00, 200.00) | 200.00 (150.00, 200.00) | 150.00 (150.00, 200.00) | 0.267 |
| HTK cardioplegic solution (into body), median (IQR) (ml) | 1900.0 (700.0, 2400.0) | 1900.0 (700.0, 2400.0) | 1900.0 (700.0, 2500.0) | 0.294 |
| HTK Cardioplegic solution (all), median (IQR) (ml) | 2000.0 (2000.0, 2500.0) | 2000.0 (2000.0, 2500.0) | 2000.0 (2000.0, 2500.0) | < 0.001^*^ |
| The total liquid infusion volume, median (IQR) (ml) | 4350.0 (3450.0, 5150.0) | 4350.0 (3400.0, 5100.0) | 4400.0 (3500.0, 5450.0) | 0.006^*^ |
| Liquid balance, median (IQR) (ml) | 700.0 (150.0, 1150.0) | 750.0 (250.0, 1200.0) | 500.0 (-150.0, 1050.0) | < 0.001^*^ |
| pRBC transfusion during surgery, median (IQR) (U) | 2.50 (0.00, 4.00) | 2.00 (0.00, 4.00) | 4.00 (2.00, 6.00) | < 0.001^*^ |
| FFP transfusion during surgery, median (IQR) (U) | 4.80 (0.00, 5.60) | 4.60 (0.00, 5.30) | 5.00 (0.00, 6.60) | < 0.001^*^ |
| PLT transfusion during surgery, median (IQR) (U) | 0.00 (0.00, 4.00) | 0.00 (0.00, 3.00) | 0.00 (0.00, 4.00) | < 0.001^*^ |
| Lowest core temperature, median (IQR) (℃) | 32.20 (31.40, 33.00) | 32.30 (31.50, 33.00) | 32.00 (31.00, 33.00) | < 0.001^*^ |
| Body temperature after perfusion, median (IQR) (℃) | 36.20 (36.00, 36.50) | 36.20 (36.00, 36.50) | 36.20 (36.00, 36.50) | 0.433 |
| Basic ACT, median (IQR) (s) | 119.00 (110.00, 129.00) | 119.00 (109.00, 129.00) | 119.00 (111.00, 130.00) | 0.299 |
| Heparinized ACT, median (IQR) (s) | 575.00 (503.00, 721.25) | 575.00 (504.00, 713.50) | 574.00 (500.00, 734.00) | 0.911 |
| Neutralizd ACT, median (IQR) (s) | 121.00 (112.00, 132.00) | 121.00 (112.00, 131.00) | 121.00 (111.00, 133.00) | 0.255 |
| Hgb after perfusion, median (IQR) (g/L) | 103.00 (96.00, 111.00) | 103.00 (97.00, 112.00) | 100.00 (94.00, 108.00) | < 0.001^*^ |
| Hematocrit after perfusion, median (IQR) (L/L) | 0.31 (0.29, 0.33) | 0.31 (0.29, 0.33) | 0.30 (0.29, 0.33) | < 0.001^*^ |
| SBP after perfusion, median (IQR) (mmHg) | 110.00 (102.00, 120.00) | 111.00 (103.00, 120.00) | 109.00 (100.00, 118.00) | 0.001^*^ |
| DBP after perfusion, median (IQR) (mmHg) | 63.00 (57.00, 70.00) | 64.00 (57.00, 70.00) | 61.00 (54.00, 67.00) | < 0.001^*^ |
| MAP after perfusion, median (IQR) (mmHg) | 78.67 (72.67, 85.33) | 79.50 (73.33, 86.00) | 77.33 (70.67, 83.00) | < 0.001^*^ |

Data are presented as median (interquartile range) or number (%) and statistical analysis was performed with Mann–Whitney U test, Pearson or Fisher Chi-square test. ^*^ means p-value < 0.05 when comparing patients with and without acute kidney injury following cardiac surgery. ^1^The definition of “Weight of the intervention” is consistent with that in EUROSCORE II risk estimator. ACEI, angiotensin-converting-enzyme inhibitor; ACT, activated clotting time; AKI, acute kidney injury; ALT, alanine aminotransferase; APTT, activated partial thrombin time; ARB, angiotensin II receptor blocker; ASA, American Society of Anesthesiologists; AST, aspartate aminotransferase; BMI, body mass index; CABG, coronary artery bypass grafting; CCS, Canadian Cardiovascular Society; Cl, chloride; CPB, cardiopulmonary bypass; DBP, diastolic blood pressure; FFP, fresh frozen plasma; HCT, hematocrit; Hgb, hemoglobin; HTK, histidine-tryptophane-ketoglutarate; INR, international normalized ratio; IV, intravenous; IVST, interventricular septal thickness; LVEDD, left ventricular end-diastolic diameter; LVEF, left ventricular ejection fraction; LVPWT, left ventricular posterior wall thickness; MAP, mean arterial pressure; MI, myocardial infarction; MPAD, inner diameter of main pulmonary artery;mPAP, mean pulmonary arterial pressure; NYHA, New York Heart Association; OHA, oral hypoglycemic agents; PLT, platelet; pRBC, packed red blood cell; RBC, red blood cell count; RRT, renal replacement therapy; RVD, right ventricular diameter; SBP, systolic blood pressure; Scr, serum creatinine; WBC, white blood cell count.


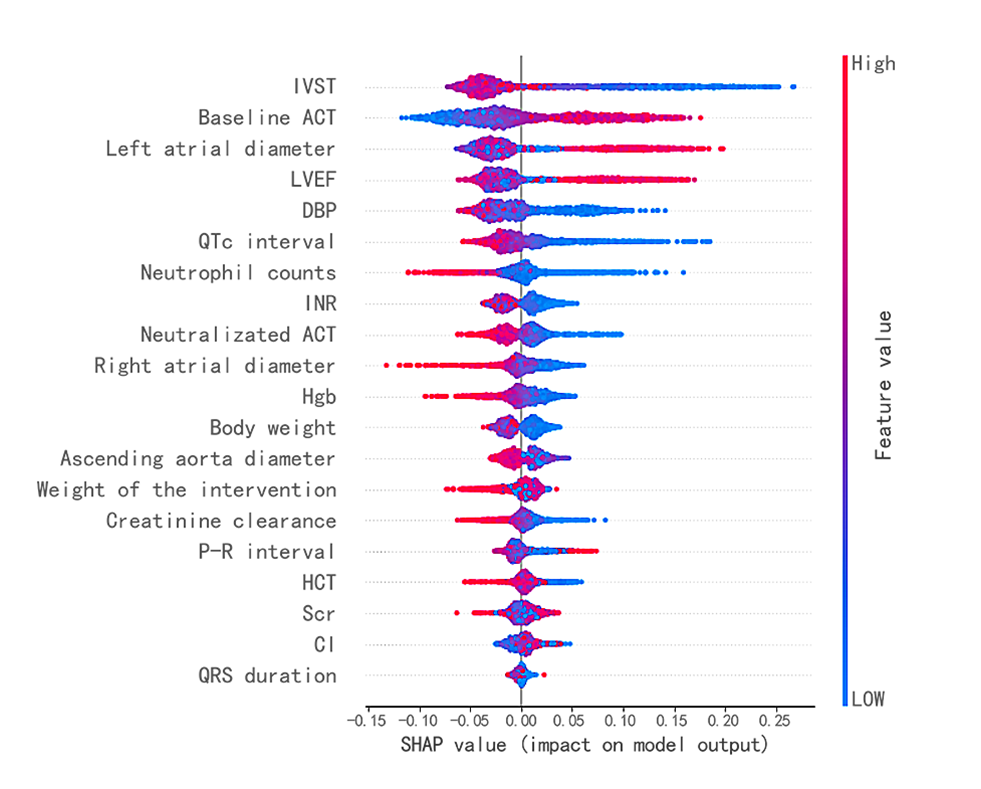


**Supplementary Fig. 1**. **SHAP summary plot of SHAP values distribution for top RF-20 features in the dataset.** The plot depicts the relative importance, impact, and contribution of different features on the running output of RF model. Each point on the summary plot is a SHapley value for a feature and an instance (i.e., a single patient encounter in this case). The color represents the high (red) to low (blue) values of the feature. The position of each point on the x-axis (i.e., the actual SHAP value) shows the impact that feature has on the classification model’s prediction for a given instance. Mathematically, a patient with a higher (absolute) SHAP value has a higher AKI risk. IVST, interventricular septal thickness; ACT, activated clotting time; LVEF, left ventricular ejection fraction; DBP, diastolic blood pressure; INR, international normalized ratio; Hgb, hemoglobin; HCT, hematocrit; Scr, serum creatinine; Cl, chloride; AKI, acute kidney injury; SHAP, SHapley Additive exPlanation; RF, random forest.

**
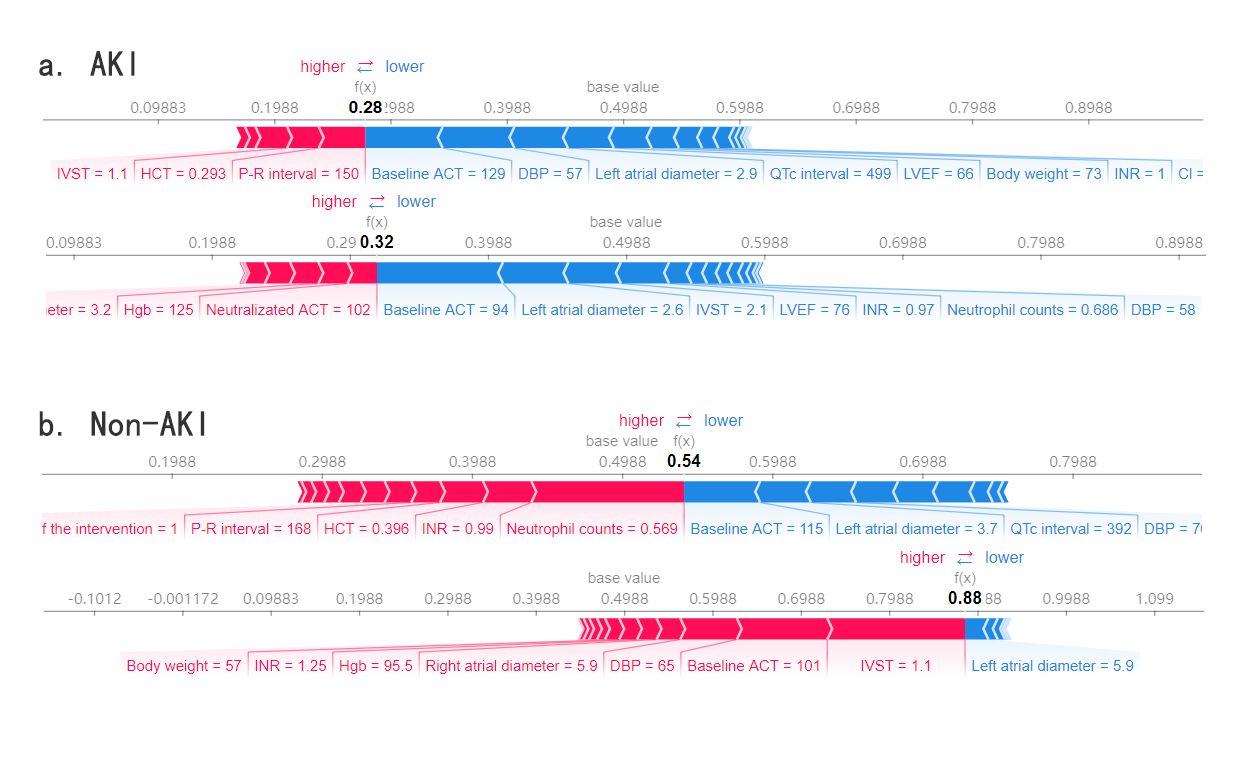
**

**Supplementary Fig. 2**. **SHAP feature importance metrics for 4 patients that were incorrectly predicted as Non-AKI (a) or AKI (b).** The base value—the mean of the model output (log-odds) over the training dataset—is 0.4988. Output values (bold), expressed as log odds ratio of probability of AKI to probability of Non-AKI (i.e., log ($\frac{P(aki)}{1-P(aki)}$)), that are low (0.28, 0.32) in AKI patients (a) and high (0.54, 0.88) in Non-AKI patients (b). Red bars indicate that the feature value is increasing the probability of AKI while blue bars indicate that the feature is decreasing it, along with the size of the bars depicting each feature’s contribution to the model’s output. AKI, acute kidney injury; IVST, interventricular septal thickness; HCT, hematocrit; ACT, activated clotting time; DBP, diastolic blood pressure; LVEF, left ventricular ejection fraction; INR, international normalized ratio; Cl, chloride; Hgb, hemoglobin.


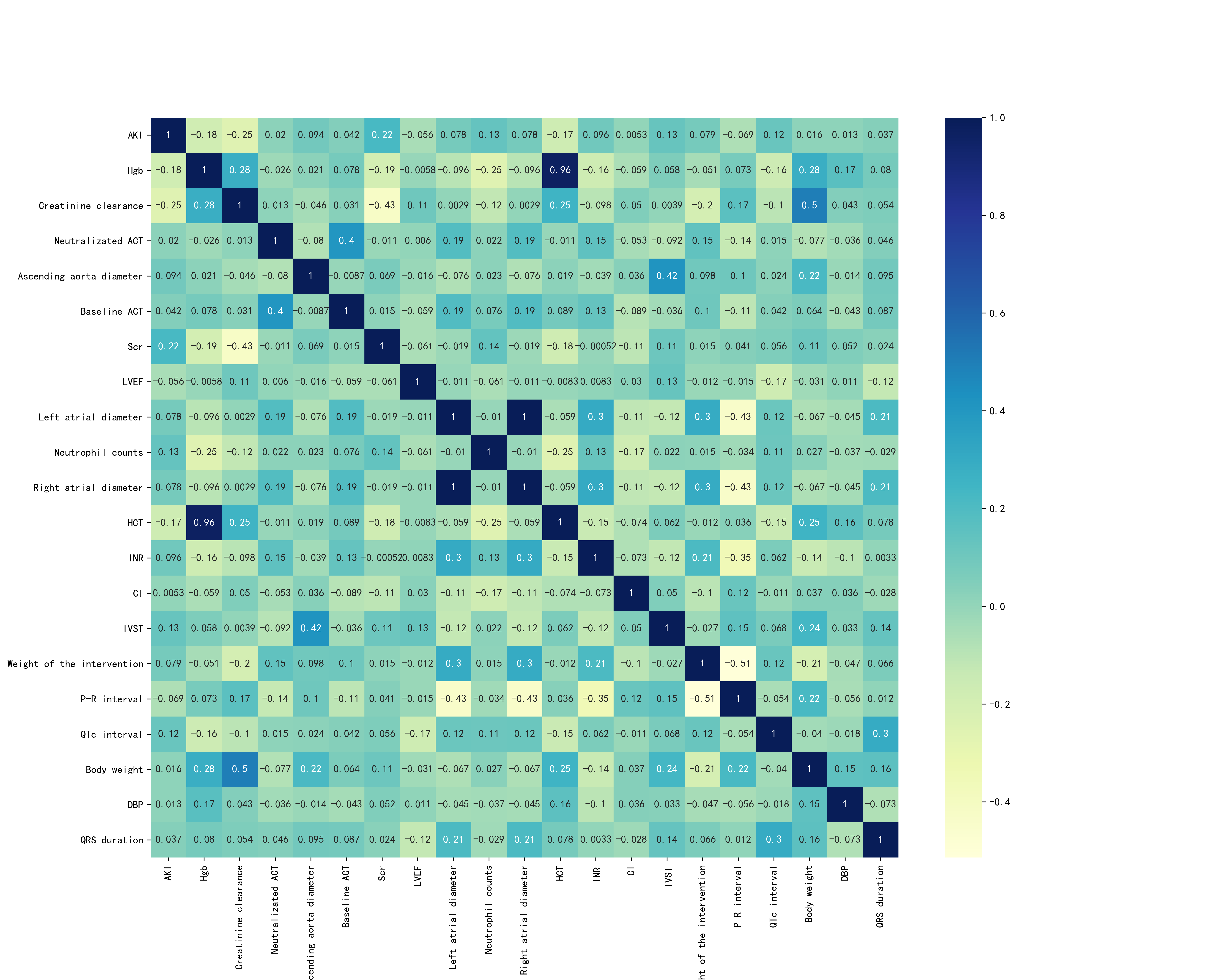


**Supplementary Fig. 3**. **Correlation heatmap analysis between risk factors and AKI.** Spearman correlation coefficient values are presented by plotting heatmap. AKI, acute kidney injury; Hgb, hemoglobin; ACT, activated clotting time; Scr, serum creatinine; LVEF, left ventricular ejection fraction; HCT, hematocrit; INR, international normalized ratio; Cl, chloride; IVST, interventricular septal thickness; DBP, diastolic blood pressure.
